# Supplementary material for: TcrXY is an acid-sensing two-component transcriptional regulator of Mycobacterium tuberculosis required for persistent infection
Source: Nat Commun. 2024 Feb 22;15:1615. doi: 10.1038/s41467-024-45343-7 (PMC10883919; doi:10.1038/s41467-024-45343-7)

Supplementary Information

TcrXY is an acid-sensing two-component transcriptional regulator of *Mycobacterium tuberculosis* required for persistent infection

Miljan Stupar, Lendl Tan, Edward D. Kerr, Christopher J. De Voss, Brian M. Forde, Benjamin L. Schulz and Nicholas P. West.

Supplementary Figures 1-11

Supplementary Table 1

Supplementary Methods

Supplementary References

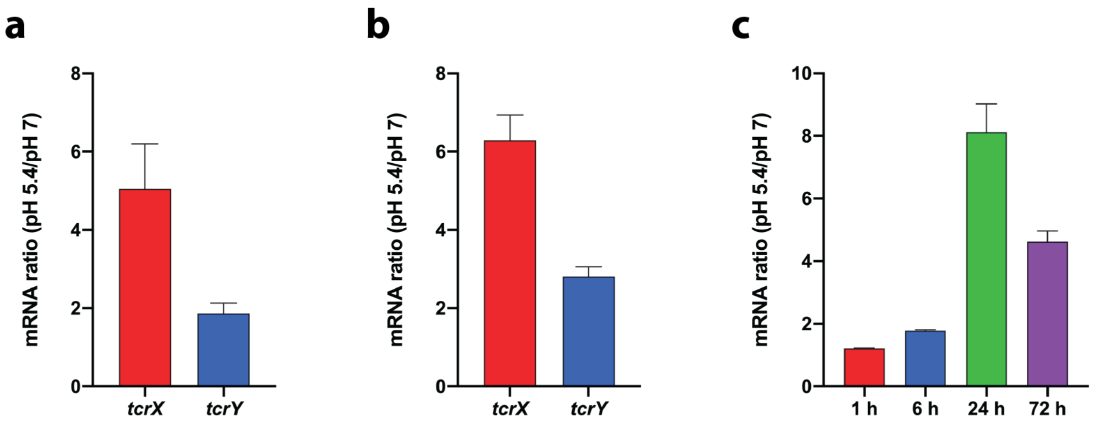

Supplementary Figure 1: *tcrXY* mRNA is induced at acidic pH.

a, *tcrXY* is induced in Mtb cultured in acidified minimal media. H37Rv was cultured in pH 7 or pH 5.4 minimal media for 24 hours and mRNA levels compared by qRT-PCR. Each bar represents the mean fold-change from four technical replicates. Error bars denote SD. **b**, *tcrXY* is induced in Mtb cultured in acidified 7H9 complete media. H37Rv was cultured in pH 7 or pH 5.4 nutrient-rich media for 24 hours and mRNA levels compared by qRT-PCR. Each bar represents the mean fold-change from four technical replicates. Error bars denote SD. **c**, *tcrX* mRNA levels reach their maximum after 24 h in acidic pH. H37Rv was cultured in pH 7 or pH 5.4 minimal media for the indicated time point and mRNA levels compared by qRT-PCR. Each bar represents the mean fold-change from four technical replicates. Error bars denote SD.

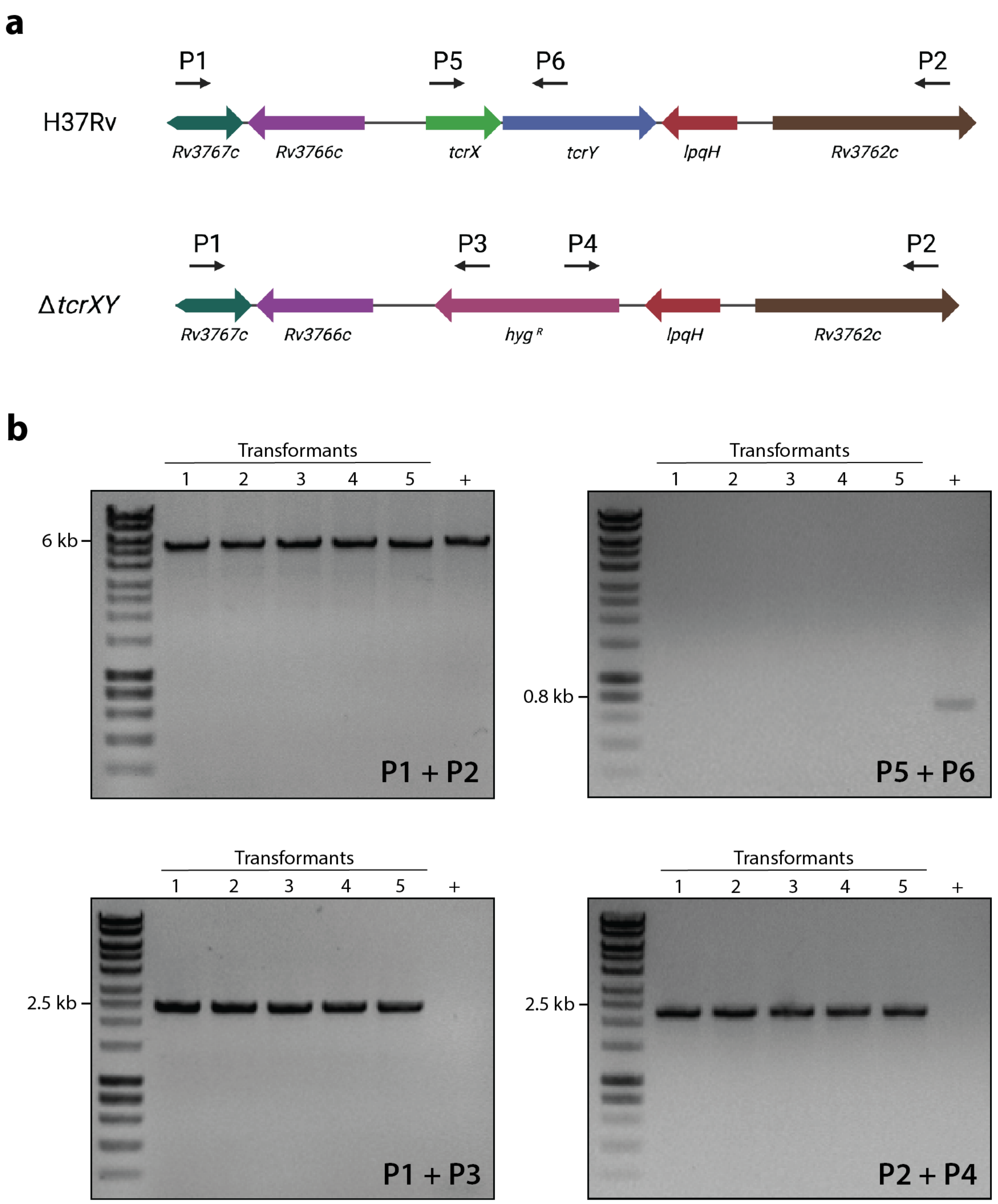

Supplementary Figure 2: Screening of colony transformants for allelic replacement of *tcrXY* in Mtb H37Rv.

a, Schematic of *tcrXY* locus depicting genomic architecture in Mtb H37Rv or expected Δ*tcrXY* mutant. Primer sets used for screening and confirmation of successful allelic exchange of a hygromycin resistance cassette (*hyg*^{R}) are shown. **b**, PCR screen using gDNA extracted from five colony transformants for successful allelic replacement of the *tcrXY* operon. In each image, lanes marked 1-5 represents the five corresponding colony transformants. "+" represents gDNA extracted from wild-type H37Rv. Expected products: P1+P2 (H37Rv: 5514-bp; Δ*tcrXY*: 5226-bp); P5+P6 (H37Rv: 763-bp; Δ*tcrXY*: none); P1+P3 (H37Rv: none; Δ*tcrXY*: 2353-bp); P2+P4 (H37Rv: none; Δ*tcrXY*: 2182-bp). Transformant "1" was the mutant used for all subsequent experiments described in this study.

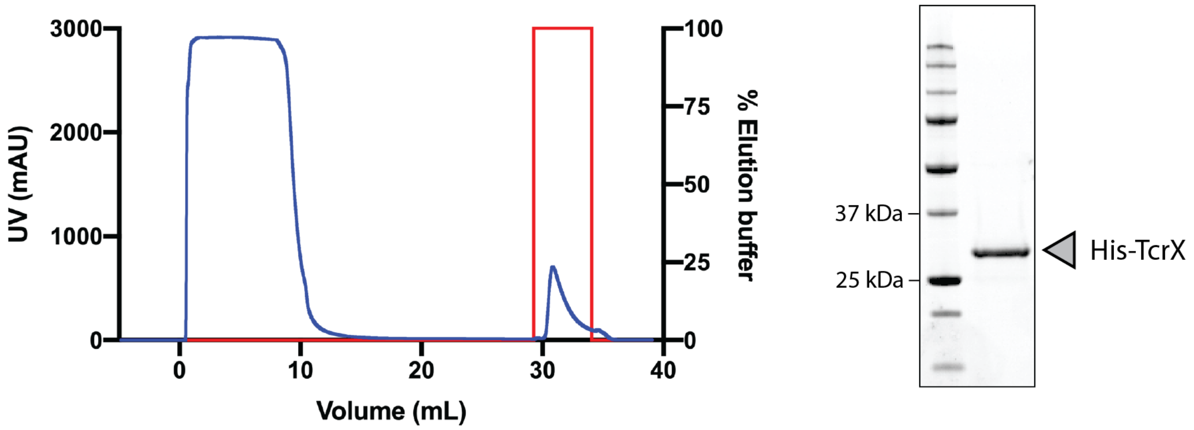

Supplementary Figure 3: Purification of Mtb TcrX.

Purification of 10xHis-TcrX by immobilised metal affinity chromatography. Graph (left) depicts UV chromatogram (blue) demonstrating successful purification of 10xHis-TcrX, using a HiTrap TALON crude column on an ÄKTApure fast-protein liquid chromatography system. The column was washed extensively, and protein eluted off the column using Elution buffer (red). Image (right) demonstrates purity of 10xHis-TcrX by 12% SDS-PAGE. Theoretical molecular weight, 31 kDa.

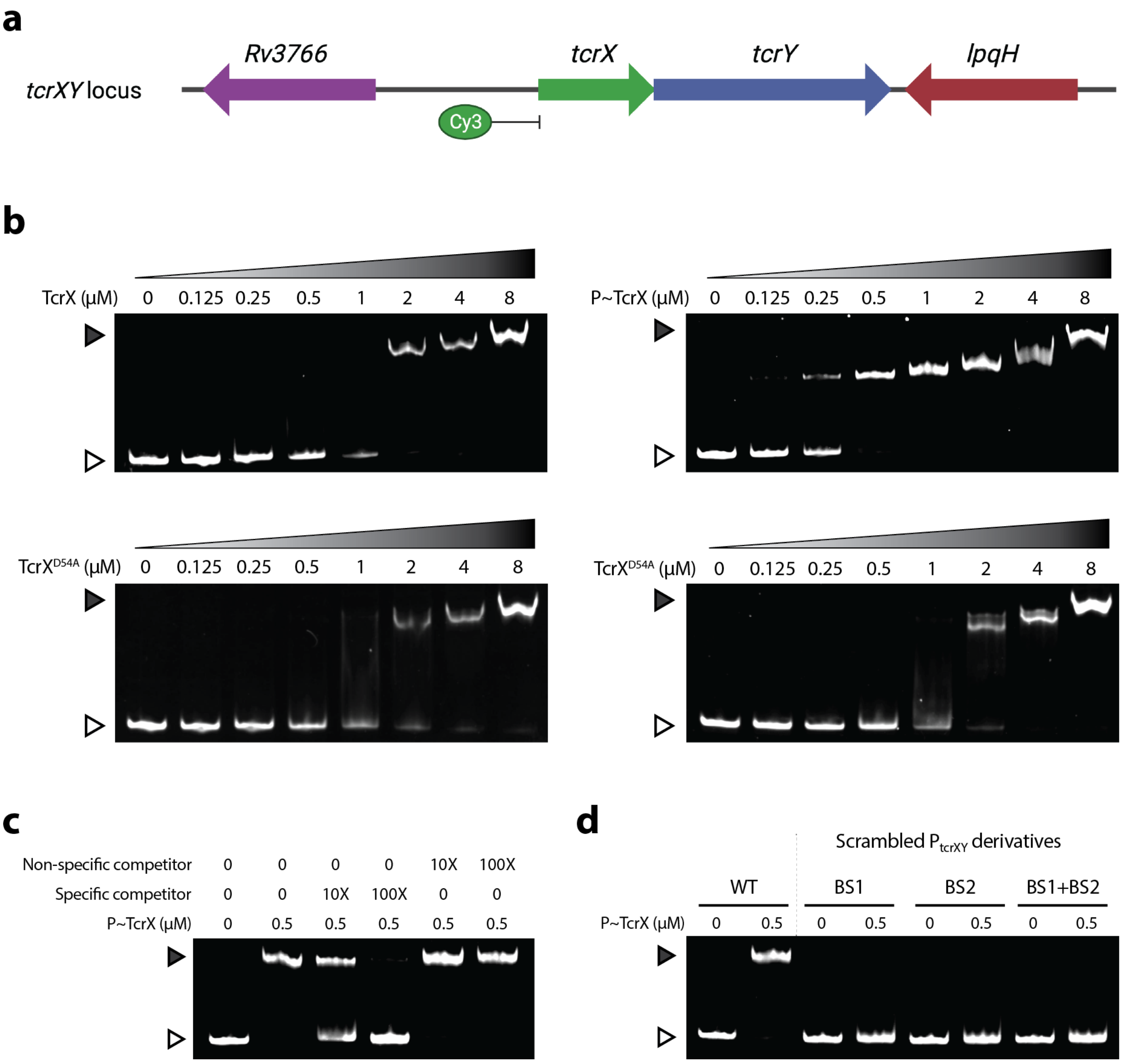

Supplementary Figure 4: A 7-bp mirror repeat in P$_{tcrXY}$ is required for TcrX binding. **a**, Schematic representation of the *tcrXY* locus and the location of the P$_{tcrXY}$ fragment. **b,** TcrX phosphorylation increases its affinity for P$_{tcrXY}$. Top left: EMSA of unphosphorylated TcrX against P$_{tcrXY}$. Top right: EMSA of phosphorylated TcrX (P~TcrX) against P$_{tcrXY}$. Bottom left: EMSA of phosphorylation-deficient TcrX (TcrX^{D54A}) against P$_{tcrXY}$. Bottom right: EMSA of TcrX^{D54A} against P$_{tcrXY}$, where an attempt was made to phosphorylate it. Increasing TcrX concentrations are depicted with each lane, against a constant quantity of DNA (10 nM). **c**, Specificity of the P~TcrX-P$_{tcrXY}$ interaction. Phosphorylated TcrX was incubated with labelled P$_{tcrXY}$, with either a 10X or 100X molar excess of unlabelled P$_{tcrXY}$ or unlabelled non-specific DNA. **d**, Sequence scrambling of a 7-bp repeat in P$_{tcrXY}$ abolishes TcrX-DNA binding. Phosphorylated TcrX was incubated with labelled P$_{tcrXY}$ variants, and their interactions assessed with an EMSA. In b-d, open arrow, free DNA; filled arrow, TcrX-DNA complex.

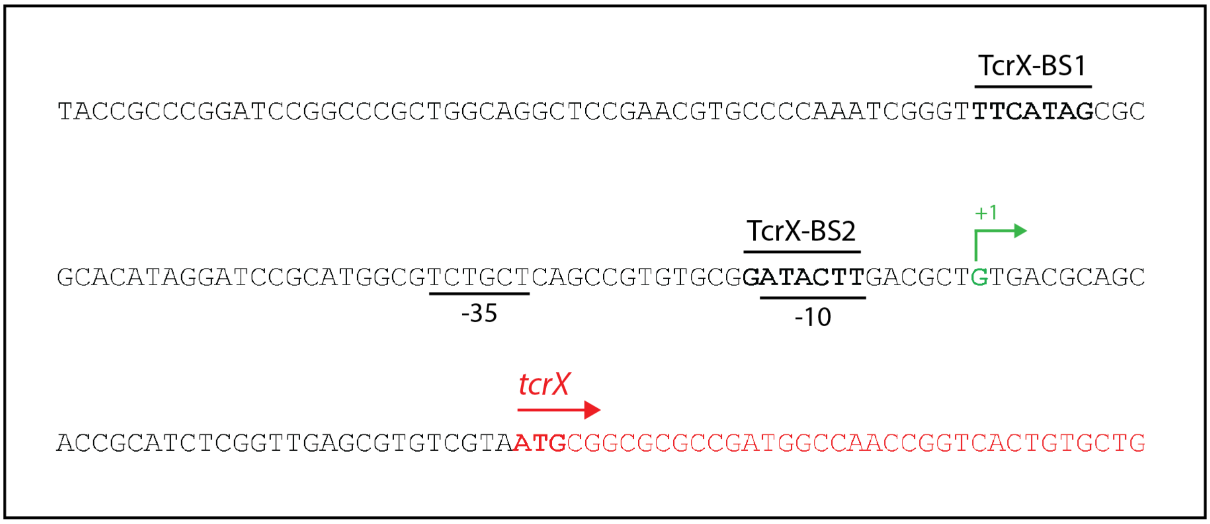

Supplementary Figure 5: Promoter region of *tcrX* in Mtb H37Rv.
Diagram depicting proposed *tcrXY* promoter structure. The putative TcrX binding site (TcrX-BS) is a mirror repeat spaced 42-bp apart, initially proposed by Bhattacharya and Das (2011)[1] but incorrectly described as an inverted repeat. The putative transcription start site (+1) was determined from Shell et al. (2015)[2]. Putative -35 and -10 promoter sites and ATG translational start site are indicated accordingly.

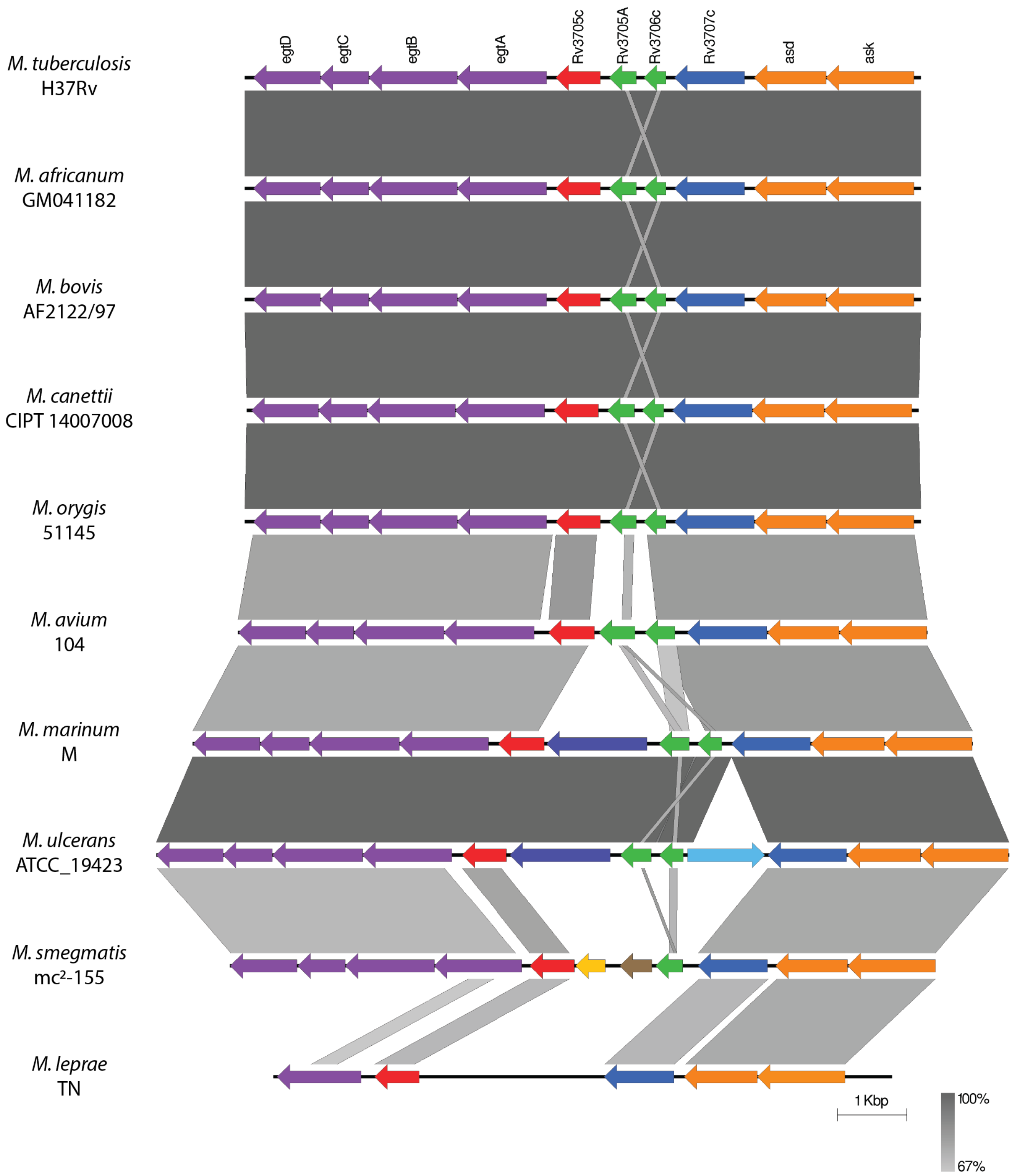

Supplementary Figure 6: Synteny in the *Rv3706c-Rv3705A* locus.

BLASTn alignment depicts the level of nucleotide sequence conservation (grey shading), amongst *Rv3706c-Rv3705A* (green), *Rv3705c* (red), *Rv3707c* (blue), ergothioneine biosynthesis cluster (purple), aspartate catabolism gene cluster (orange). Reference genomes were acquired from NCBI: *M. tuberculosis* H37Rv (NC_000962.3), *M. africanum* GM041182 (FR878060.1), *M. bovis* AF2122/97 (LT708304.1), *M. canettii* CIPT14007008 (FO203508.1), *M. orygis* 51145 (NZ_CP063804.1), *M. avium* 104 (CP000479.1), *M. marinum* M (CP000854.1), *M. ulcerans* ATCC_19423 (NZ_CP092429.2), *M. smegmatis* mc^2-155 (CP001663.1), *M. leprae* TN (AL450380.1). Figure was constructed using EasyFig[3].

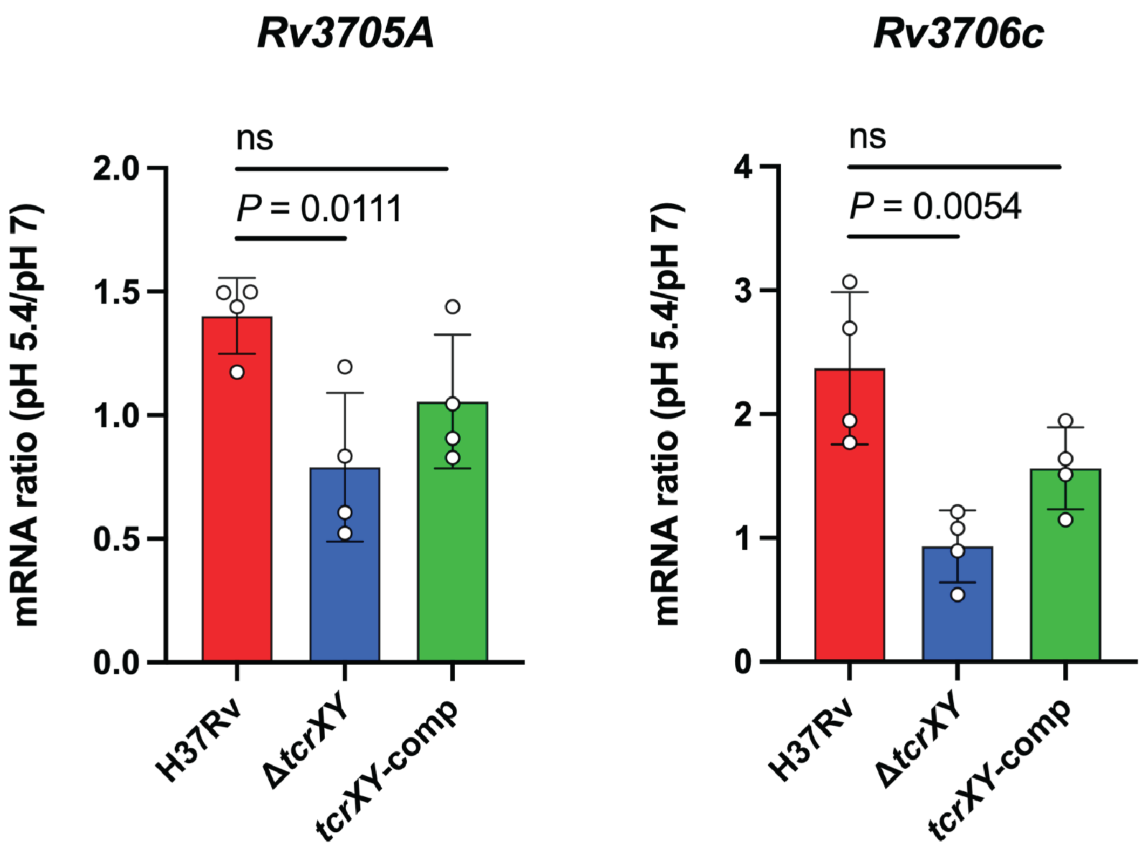

Supplementary Figure 7: qRT-PCR confirmation of *Rv3706c* and *Rv3705A* expression. Indicated strains were cultured in pH 7 or pH 5.4 minimal media for 24 hours and mRNA levels compared by qRT-PCR. Each bar represents the mean fold-change from four biological replicates (open circle). Error bars denote SD. Statistical comparisons were performed using a two-tailed t-test. ns, not significant.

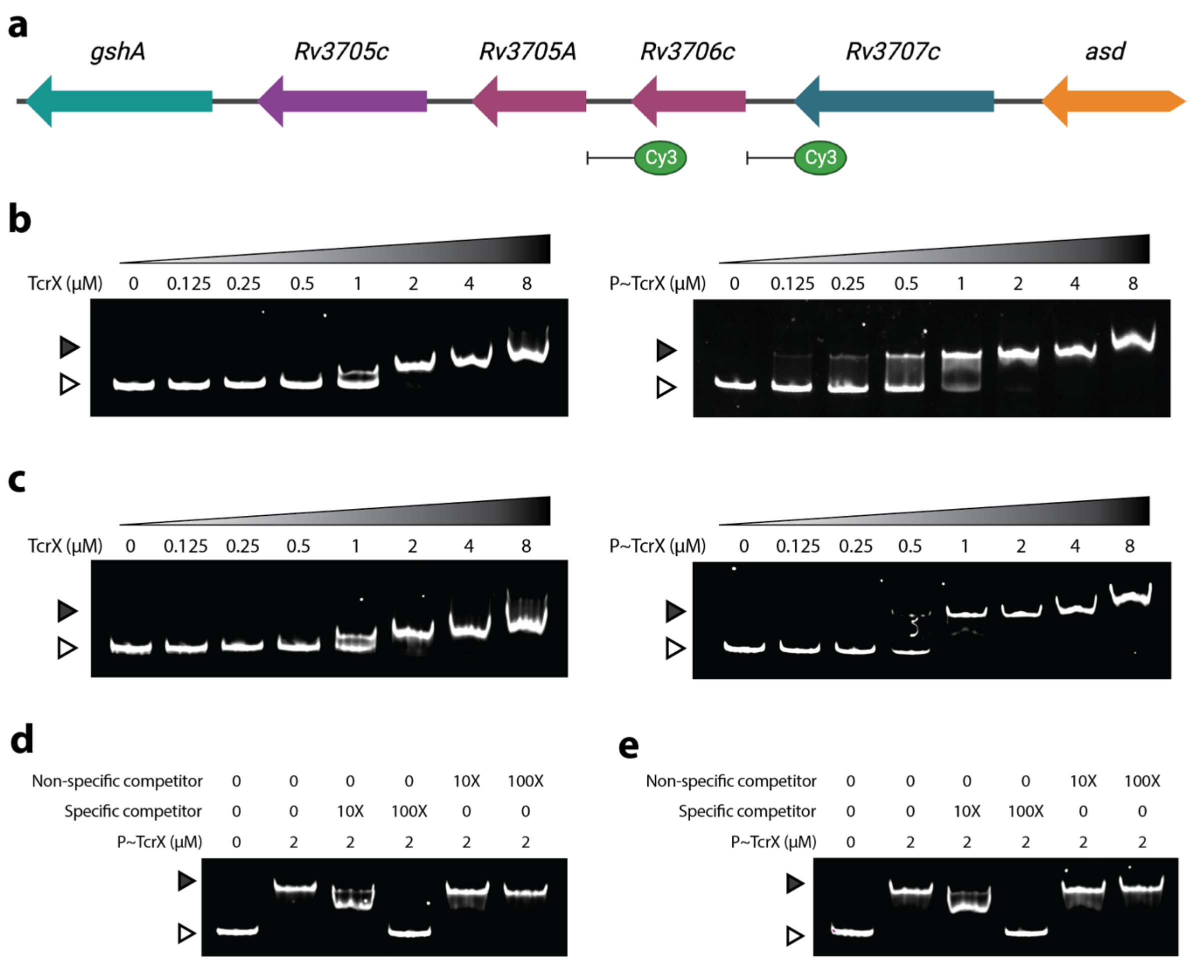

Supplementary Figure 8: TcrX directly regulates *Rv3706c* and *Rv3705A*.
a, Schematic representation of the *Rv3706c-Rv3705A* locus and the location of the $P_{Rv3706c}$ and $P_{Rv3705A}$ fragment (Cy3-labelled). **b,** TcrX directly interacts with $P_{Rv3706c}$. Left: EMSA of unphosphorylated TcrX against $P_{Rv3706c}$. Right: EMSA of phosphorylated TcrX (P~TcrX) against $P_{Rv3706c}$. Increasing TcrX concentrations are depicted with each lane, against a constant quantity of DNA (10 nM). **c,** TcrX directly interacts with $P_{Rv3705A}$. Left: EMSA of unphosphorylated TcrX against $P_{Rv3705A}$. Right: EMSA of phosphorylated P~TcrX against $P_{Rv3705A}$. Increasing TcrX concentrations are depicted with each lane, against a constant quantity of DNA (10 nM). **d**, Specificity of the P~TcrX-$P_{Rv3706c}$ interaction. Phosphorylated TcrX was incubated with 10 nM labelled $P_{Rv3706c}$ with either a 10X or 100X molar excess of unlabelled specific DNA or unlabelled non-specific DNA. **e**, Specificity of the P~TcrX-$P_{Rv3705A}$ interaction. Phosphorylated TcrX was incubated with 10 nM labelled $P_{Rv3705A}$ with either a 10X or 100X molar excess of unlabelled specific DNA or unlabelled non-specific DNA.

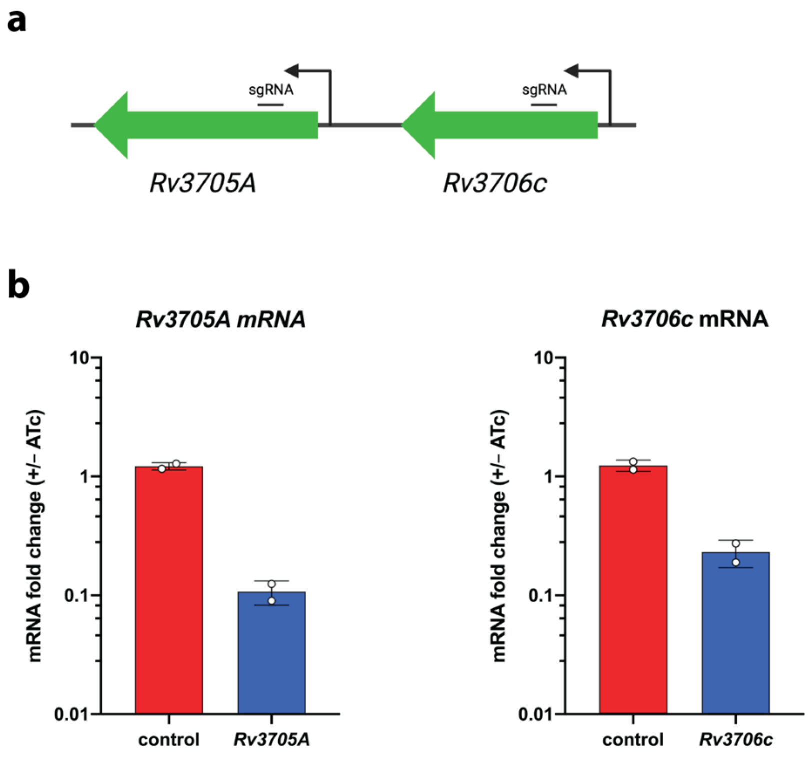

Supplementary Figure 9: Construction of CRISPRi-*Rv3706c* and CRISPRi-*Rv3705A*.
a, Silencing of *Rv3706c and Rv3705A* using CRISPRi, to generate the CRISPRi strains, CRISPRi-*Rv3706c* and CRISPRi-*Rv3705A*. sgRNA target site is indicated. **b**, *Rv3706c* and *Rv3705A* knockdown using CRISPRi. The CRISPRi strains were cultured in the presence or absence of ATc in 7H9 media for 72 hours and mRNA levels compared by qRT-PCR. Each bar represents the mean fold-change from two biological replicates (open circle). Error bars denote SD.

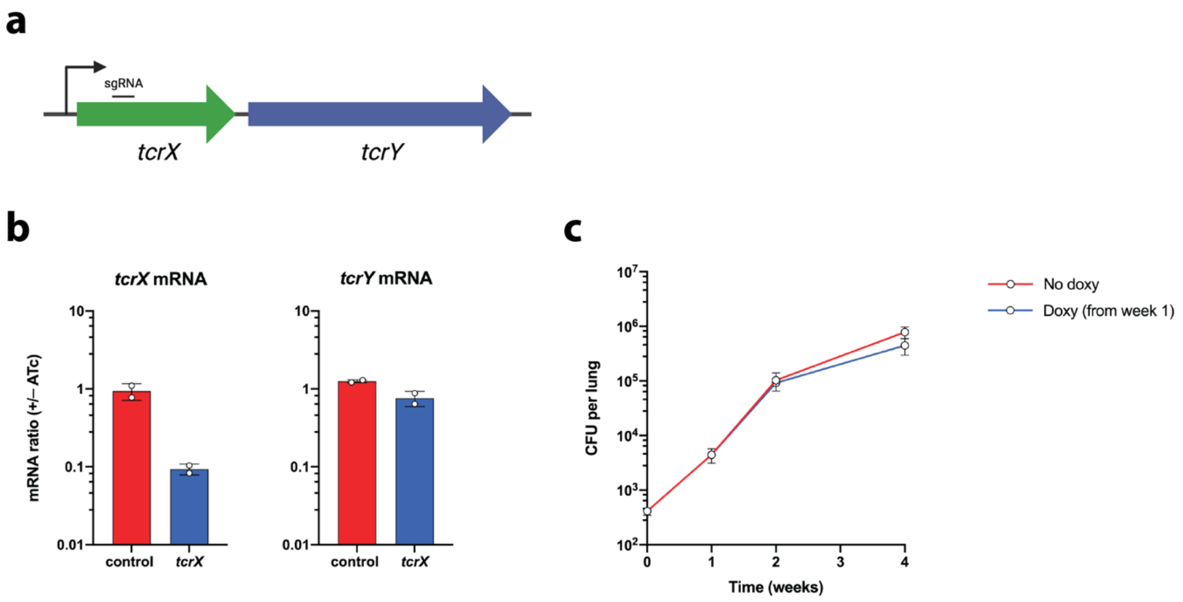

Supplementary Figure 10: Construction of VICE-*tcrX* and growth during acute lung infection.

a, Silencing of *tcrX* using CRISPRi, to generate the strain, VICE-*tcrX*. sgRNA target site is indicated. **b**, *tcrXY* knockdown using CRISPRi. The CRISPRi-*tcrX* mutant was cultured in the presence or absence of ATc in 7H9 media for 72 hours and mRNA levels compared by qRT-PCR. Each bar represents the mean fold-change from two biological replicates (open circle). Error bars denote SD. **c**, CRISPRi-mediated *tcrX* silencing in Mtb during acute lung infection. Female C57BL/6 mice were infected with < 500 CFU of CRISPRi-*tcrX* by low-dose aerosol, and the infectious dose was confirmed from two mice on day 1. Each time point then represents mean recovered CFU from each lung from four mice, with error bars denoting SD. CRISPRi was induced by administration of doxycycline (1 mg/mL in 5% sucrose, delivered as drinking water *ad libitum*) from week 1 post-infection. Statistical analysis between no doxy and doxy groups was performed using an unpaired two-tailed t-test at week 2 and week 4, with no significant comparisons observed.

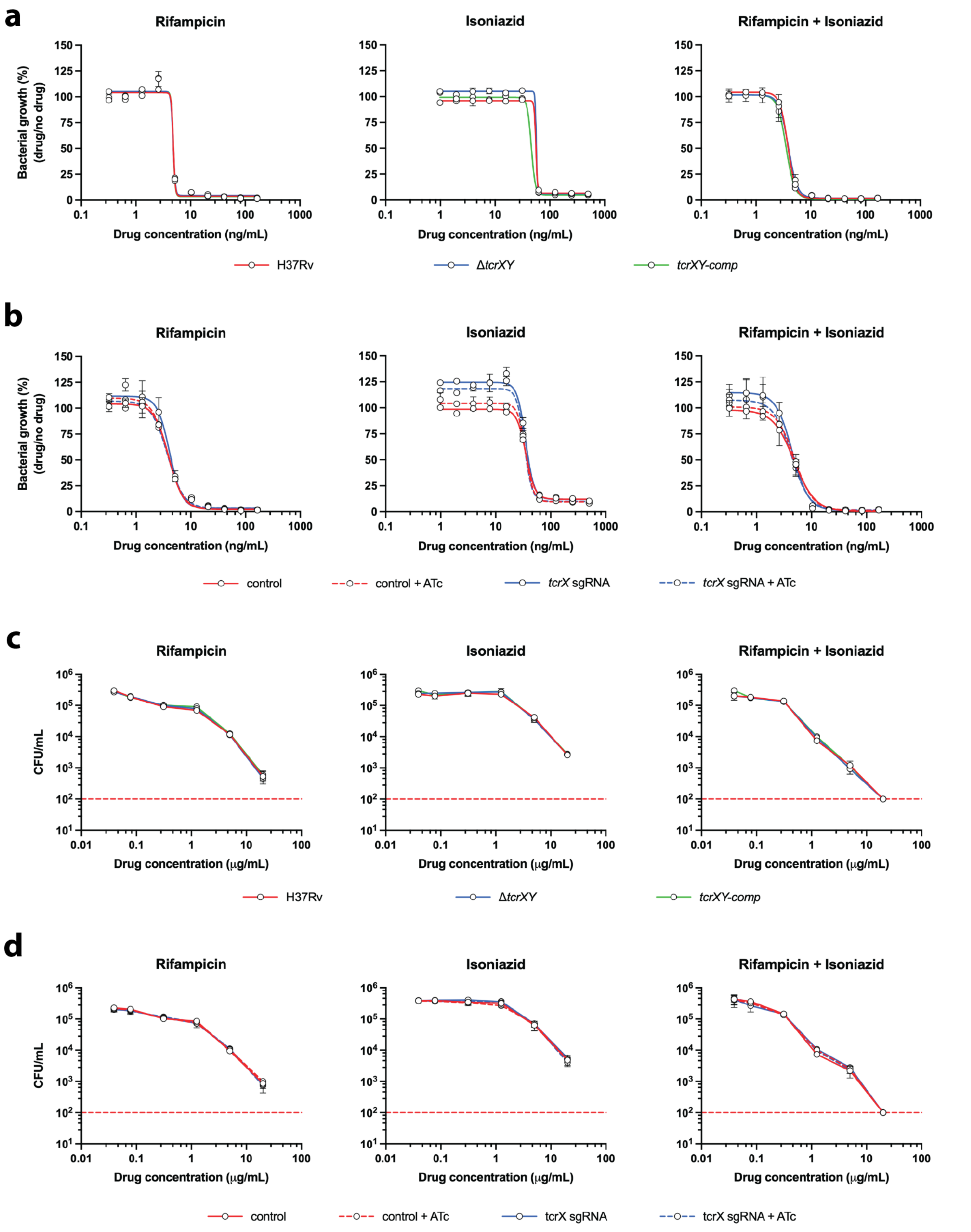

Supplementary Figure 11: Drug susceptibility profiling of Mtb *tcrXY* mutants.

a, Dose-response curves to rifampicin, isoniazid, or a combination of rifampicin and isoniazid, of H37Rv, Δ*tcrXY* and *tcrXY*-comp, in standard nutrient-rich 7H9 liquid culture. Data is shown as mean with SD from three parallel cultures. **b**, Dose-response curves to rifampicin, isoniazid, or a combination of rifampicin and isoniazid, of CRISPRi-*tcrX* and an empty-vector control, in standard nutrient-rich 7H9 liquid culture. Data is shown as mean with SD from three parallel

cultures. Cultures were pre-depleted with anhydrotetracycline (ATc) for 3 days prior, and maintained in the culture medium for the duration of the assay. **c**, Quantification of H37Rv, Δ*tcrXY* and *tcrXY*-comp killing by rifampicin, isoniazid, or a combination of rifampicin and isoniazid, in standard nutrient-rich 7H9 liquid culture. Data is shown as mean CFU with SD from three parallel cultures. **d**, Quantification of CRISPRi-tcrX killing by rifampicin, isoniazid, or a combination of rifampicin and isoniazid, in standard nutrient-rich 7H9 liquid culture. Data is shown as mean CFU with SD from three parallel cultures. Cultures were pre-depleted with ATc for 3 days prior, and maintained in the culture medium for the duration of the assay.

Supplementary Table 1: Description of plasmids used in this study.

Plasmid name	Description	Source
pMV206	Promoterless *E. coli*-mycobacterial shuttle vector; MCS; OriE; OriM; Km^R	Stover et al. (1991)[4]
pMV306	Promoterless *E. coli*-mycobacterial shuttle vector; MCS; OriE; L5 int; Km^R	Stover et al. (1991)[4]
pMV306GI	Promoterless *E. coli*-mycobacterial shuttle vector; MCS; OriE; Giles int; Ntc^R	This study
**Reporter constructs**		
pDual206	pMV206 containing promoter-less *mCherry* and P_{rpoB}-*gfp* promoter-reporter fusion	This study
pDual306	pMV306 containing promoter-less *mCherry* and P_{rpoB}-*gfp* promoter-reporter fusion	This study
pDual306-control	pDual306 containing $P_{control}$ promoter-reporter fusion	This study
pDual206-*tcrXY*	pDual206 containing P_{tcrXY} promoter-reporter fusion	This study
pDual306-*tcrXY*	pDual306 containing P_{tcrXY} promoter-reporter fusion	This study
pDual306-*tcrXY* (BS1 scramble)	pDual306 containing P_{tcrXY} (BS 1 scrambled) promoter-reporter fusion	This study
pDual306-*tcrXY* (BS2 scramble)	pDual306 containing P_{tcrXY} (BS 2 scrambled) promoter-reporter fusion	This study
pDual306-*tcrXY* (BS1+2 scramble)	pDual306 containing P_{tcrXY} (BS 1+2 scrambled) promoter-reporter fusion	This study
pDual206-*Rv3706c*	pDual206 containing $P_{Rv3706c}$ promoter-reporter fusion	This study
pDual306-*Rv3706c*	pDual306 containing $P_{Rv3706c}$ promoter-reporter fusion	This study
**Genetic knockouts**		
pJV53	Recombineering vector encoding Che9c mycobacteriophage recombinases; OriE; OriM; Km^R	van Kessel and Hatfull (2007)[5]
pYUB854	Mycobacterial suicide vector; OriE; Hyg^R; two MCS	van Kessel and Hatfull (2007)[5]
pYUB854-*tcrXY*	pYUB854 encoding 1-kb *tcrXY* flanking sequences	This study
**Complementation constructs**		
pMV306-*tcrXY*	pMV306 containing *tcrXY* coding region under the control of P_{tcrXY}	This study
pMV306-*tcrX(D54A)Y*	pMV306 containing *tcrXY* coding region, with TcrX(D54A) substitution, under the control of P_{tcrXY}	This study
pMV306-*tcrXY(H265Q)*	pMV306 containing *tcrXY* coding region, with TcrY(H256Q) substitution, under the control of P_{tcrXY}	This study
**CRISPRi constructs**		
PLJR965	Mycobacterial CRISPR interference vector, inducible TetR system, OriE; L5 int; Km^R	Rock et al. (2017)[6]
PLJR965-*tcrX*	PLJR965 containing *tcrX* sgRNA	This study
PLJR965-*Rv3706c*	PLJR965 containing *Rv3706c* sgRNA	This study
PLJR965-*Rv3705A*	PLJR965 containing *Rv3705A* sgRNA	This study
pMV306GI-*Rv3706c*$_{res}$	pMV306GI containing *Rv3706c* CRISPRi-resistant allele	This study
pMV306GI-*Rv3705A*$_{res}$	pMV306GI containing *Rv3705A* CRISPRi-resistant allele	This study

Abbreviations: OriE, *E. coli* origin of replication; OriM, mycobacterial origin of replication; Km^R, kanamycin resistance marker; Hyg^R, hygromycin resistance marker; Ntc^R, nourseothricin resistant marker; MCS, multiple cloning site; L5 int, mycobacteriophage L5 integration cassette; Giles int, Giles integration cassette.

Supplementary Methods

Cloning of mycobacterial recombineering plasmids.

Plasmid pYUB854-*tcrXY* was constructed by PCR amplifying a 1-kb region upstream of *tcrX* from H37Rv gDNA using primers len41 and len30, and downstream of *tcrY* using primers len33 and prw4, and cloning into XbaI and HindII sites of pYUB854, respectively. The *tcrXY* allelic exchange cassette was PCR amplified from pYUB854-*tcrXY* using primers len41 and prw4.

Cloning of complementation plasmids.

Complementation plasmid pMV306-*tcrXY* was constructed by PCR amplifying the *tcrXY* allele under the control of its endogenous promoter (P_{tcrXY}) from H37Rv gDNA using primers wpr617 and wpr619 and cloning into XbaI site of pMV306. Plasmid pMV306-*tcrX*^{D54A}*Y* was constructed by PCR using pMV306-*tcrXY* as a template with mutagenic primers wpr723 and wpr724. Plasmid pMV306-*tcrXY*^{H256Q} was constructed by PCR using pMV306-*tcrXY* as a template with mutagenic primers wpr725 and wpr726.

Cloning of reporter constructs.

Reporter constructs were all derived from vectors pDual206 or pDual306. To construct pCherry, *mCherry* was PCR amplified using primers wpr780 and wpr781 and cloned into the EcoRV site of pMV206. Reporter *gfp* was PCR amplified using primers wpr759 and wpr760 and cloned into the HindIII site of pCherry to construct pCherryGFP. The *mmaA4* transcriptional terminator from Mtb H37Rv was PCR amplified from gDNA using primers wpr865 and wpr866 and cloned into BamHI of pCherryGFP to construct pCherry-term-GFP. Finally, to construct pDual206, the *rpoB* promoter in Mtb H37Rv was PCR amplified from gDNA using primers wpr765 and wpr766 and cloned into the EcoRI site of pCherry-term-GFP. pDual306 was constructed by cloning the *mCherry*-terminator-P_{rpoB}-*gfp* transcriptional fusion construct from pDual206 using primers wpr883 and wpr604 into EcoRV/HindIII of pMV306.

Plasmids pDual206-*tcrXY* and pDual306-*tcrXY* were constructed by PCR amplifying P_{tcrXY} from H37Rv gDNA using primers wpr783 and wpr784 and cloning into XbaI of pDual206 and pDual306, respectively. Plasmids pDual306-*tcrXY*(BS1+2 scrambled), pDual306-*tcrXY*(BS1 scrambled) and pDual306-*tcrXY*(BS2 scrambled) was constructed from a custom synthesised gene block (Integrated DNA Technologies) and cloning into XbaI of pDual306. The reporter

plasmids pDual206-*Rv3706c* and pDual306-*Rv3706c* were generated by PCR amplifying $P_{Rv3706c}$ using primers wpr1454 and wpr1455 from H37Rv gDNA and cloning into the XbaI site of pDual206 and pDual306, respectively.

Whole genome sequencing and analysis.

Mtb H37Rv or Δ*tcrXY* were harvested at mid-log growth phase (~ 10^9 CFU) by centrifugation and then resuspended in 5 mL of TE buffer. The cell suspensions were combined with an equal volume of chloroform:methanol (2:1) and then incubated with rocking for 45 minutes at room temperature. Samples were collected by centrifugation (2500 x *g*, 20 minutes, 4°C), supernatant discarded, and the residual pellet dried under N_2 gas. The dried pellet was resuspended in 3 mL of TE buffer, and cell lysis performed using 400 µg/mL of lysozyme (Sigma) with overnight incubation at 37°C. Sodium dodecyl sulfate (SDS) was added to a final concentration of 1%, and samples digested with 100 µg/mL of proteinase K (Qiagen) and 10 µg/mL of RNase A (Thermo Fisher) at 55°C for 3 hours. An equal volume of phenol:chloroform:isoamyl alcohol (24:24:1) was added to the cell lysate and incubated with rocking for 30 minutes. The aqueous phase was collected following centrifugation (2500 x *g*, 30 minutes, room temperature), and genomic DNA (gDNA) precipitated by the additional of 0.1 volume of 3 M sodium acetate and 1 volume of isopropanol. The pellet was washed twice with 70% ethanol, air dried, and then resuspended in 0.2 mL of TE buffer. Final gDNA clean-up was performed using a Genomic DNA Clean & Concentrator kit (Zymo Research).

Extracted gDNA was analysed by whole genome sequencing using both short- and long-read platforms. For short-read sequencing, gDNA was sequenced on the NovaSeq6000 (Illumina) using NovaSeq6000 SP kit v1.5 (2 x 150-bp paired end chemistry), in the Australian Centre for Ecogenomics at The University of Queensland. Trimmomatic (v0.36)[7] was used for filtering the raw reads, with the quality of the reads assessed before and after filtering using FastQC (v0.11.5) (https://www.bioinformatics.babraham.ac.uk/projects/fastqc/). For long-read sequencing, gDNA was loaded onto a single FLO-MIN106 flow cell using the rapid barcode sequencing kit (SQK-RBK004) as per manufacturer's recommendation with the following adjustments: the barcoded DNA was pooled without a concentration step using AMPure XP beads prior to sequencing. Barcodes and adaptors were trimmed using Porechop (v0.2.3_seqan2.1.1) (https://github.com/rrwick/Porechop). A hybrid genome assembly was generated using Flye (v2.5)[8] as implemented in MicroPIPE[9].

TcrX purification.

Full-length TcrX corresponding to amino acids 1-234 was overexpressed in *E. coli* BL21[DE3] as a 10xHis-fusion protein using a pET19b expression plasmid (Novagen). The protein was co-expressed with GroEL and GroES chaperones, expressed from plasmid pGTf2 using 20 ng/mL tetracycline as inducer. After overnight induction with 0.5 mM IPTG at 16°C (with shaking at 120 rpm), bacteria were harvested by centrifugation (4500 x *g*, 45 minutes, 4°C) and either stored at -20°C or used immediately for protein purification. Cell pellets were resuspended in Buffer A (50 mM Tris-HC pH 8, 500 mM NaCl, 10 mM imidazole, 10% glycerol) supplemented with cOmplete EDTA-free protease inhibitors (Roche) and lysed by sonication. The cell lysate was clarified by centrifugation (50,000 x g, 1 hour, 4°C) and injected onto a 1 mL HisTrap HP column (Cytiva) using an ÄKTApure fast protein liquid chromatography (FPLC) system maintained at 4°C. The column was washed extensively with Buffer A, and eluted using Buffer B (50 mM Tris-HCl pH 8, 500 mM NaCl, 300 mM imidazole, 10% glycerol). Purified 10xHis-TcrX was dialysed into Storage Buffer (50 mM Tris-HCl pH 8, 500 mM NaCl, 25% glycerol), concentrated using an Amicon Ultra-4 mL centrifugal filter (Merck), snap-frozen in liquid nitrogen and stored at -80°C. The phosphorylation-deficient variant, $TcrX^{D54A}$, was purified using the same strategy.

Electrophoretic mobility shift assays.

Purified TcrX was incubated with DNA substrates (10 nM) in binding buffer (50 mM Tris-HCl pH 7.4, 20 mM NaCl, 2 mM $MgCl_2$, 1 mM EDTA, 0.1 mg/mL BSA, 10% glycerol) in a 10 µL reaction at 25°C for 1 hour. When indicated, TcrX was phosphorylated with acetyl phosphate (Sigma Aldrich) at 25°C for 2 hours prior to performing the assay. The binding reaction was analysed on a 5% polyacrylamide gel, electrophoresed in 0.5X TBE buffer at 4°C protected from light. Cy3-labelled DNA was visualised with the Amersham imager 800 (Cytiva).

Drug susceptibility profiling.

The minimum inhibitory concentration (MIC) of rifampicin and isoniazid against Mtb was determined using a resazurin reduction microplate assay. Drugs were serially diluted across a 96-well microtiter plate in 7H9S culture media (7H9 supplemented with 10% OADC, 0.5% glycerol, 0.05% Tween-80 and 1% tryptone). All bacteria were prepared to mid-log growth phase in 7H9S medium, prior to the generation of a single cell suspension by slow centrifugation (800 x *g*, 8 min) and adjusted to $2x10^4$ CFU/mL (OD_{600} 0.001). All drugs were

dissolved in dimethyl sulfoxide (DMSO), and the final DMSO concentration was < 0.1%. CRISPRi strains were induced with 200 ng/mL ATc for 3 days prior to the assay, and ATc (200 ng/mL) was maintained in the culture medium for the duration of the assay. The plates were incubated for 6 days at 37°C, before the addition of 30 μL of a 0.02% resazurin solution and 12.5 μL of a 20% Tween-80 solution. Plates were further incubated for 24 hours to allow for colour development. Sample fluorescence was measured on a Fluorostar Omega fluorescent plate reader (BMG) (excitation 530 nm, emission 590 nm). Percent fluorescence relative to the positive control wells (bacteria without compound) minus the negative control wells (media only) was plotted for the determination of the MIC (i.e., the lowest concentration at which the percentage inhibition was 90% or greater).

To assess Mtb killing by rifampicin and isoniazid, drugs were serially diluted across a 96-well microtiter plate in 7H9 culture media. All bacterial strains were prepared to mid-log growth phase in 7H9 growth media, prior to the generation of a single cell suspension by slow centrifugation (800 x *g*, 8 min) and adjusted to $2x10^4$ CFU/mL (OD_{600} 0.001). All drugs were dissolved in DMSO, and the final DMSO concentration was < 0.1%. CRISPRi strains were induced with 200 ng/mL ATc for 3 days prior to the assay, and ATc (200 ng/mL) was maintained in the culture medium for the duration of the assay. The plates were incubated for 6 days at 37°C. Following incubation, selected wells from the microtiter plate were further serially diluted in PBS-tyloxapol, and then plated onto solid agar. CFU was quantified following incubation (3 weeks).

Supplementary References

1. Bhattacharya, M. & Das, A.K. Inverted repeats in the promoter as an autoregulatory sequence for TcrX in *Mycobacterium tuberculosis*. *Biochem Biophys Res Commun* **415**, 17-23 (2011).
2. Shell, S.S. *et al.* Leaderless transcripts and small proteins are common features of the mycobacterial translational landscape. *PLoS Genet* **11**, e1005641 (2015).
3. Sullivan, M.J., Petty, N.K. & Beatson, S.A. Easyfig: a genome comparison visualizer. *Bioinformatics* **27**, 1009-10 (2011).
4. Stover, C.K. *et al.* New use of BCG for recombinant vaccines. *Nature* **351**, 456-60 (1991).
5. van Kessel, J.C. & Hatfull, G.F. Recombineering in *Mycobacterium tuberculosis*. *Nat Methods* **4**, 147-52 (2007).
6. Rock, J.M. *et al.* Programmable transcriptional repression in mycobacteria using an orthogonal CRISPR interference platform. *Nat Microbiol* **2**, 16274 (2017).
7. Bolger, A.M., Lohse, M. & Usadel, B. Trimmomatic: a flexible trimmer for Illumina sequence data. *Bioinformatics* **30**, 2114-20 (2014).
8. Kolmogorov, M., Yuan, J., Lin, Y. & Pevzner, P.A. Assembly of long, error-prone reads using repeat graphs. *Nat Biotechnol* **37**, 540-546 (2019).
9. Murigneux, V. *et al.* MicroPIPE: validating an end-to-end workflow for high-quality complete bacterial genome construction. *BMC Genomics* **22**, 474 (2021).

Uncropped Figures

Supplementary Figure 2b
Uncropped agarose gel images demonstrating construction of ΔtcrXY

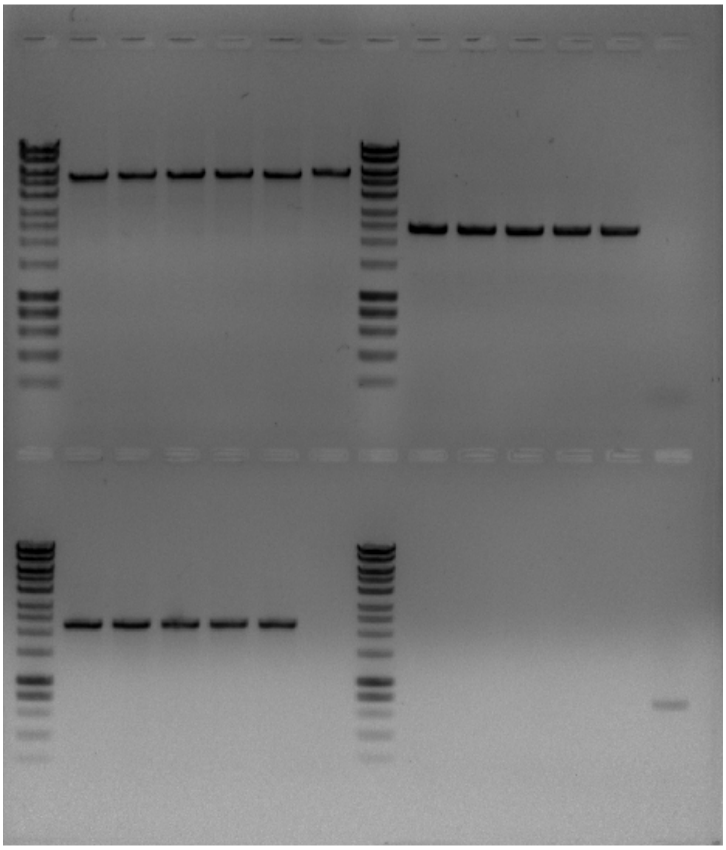

Supplementary Figure 3
Uncropped polyacrylamide gel image of purified His-TcrX.

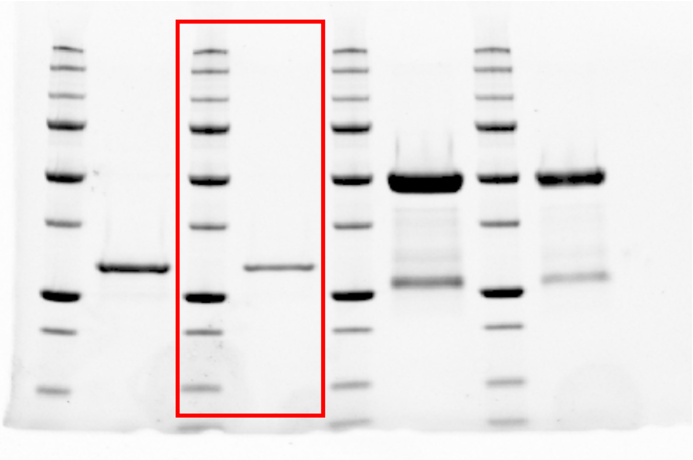

NB: Red box indicates cropped image of purified His-TcrX

Supplementary Figure 4b

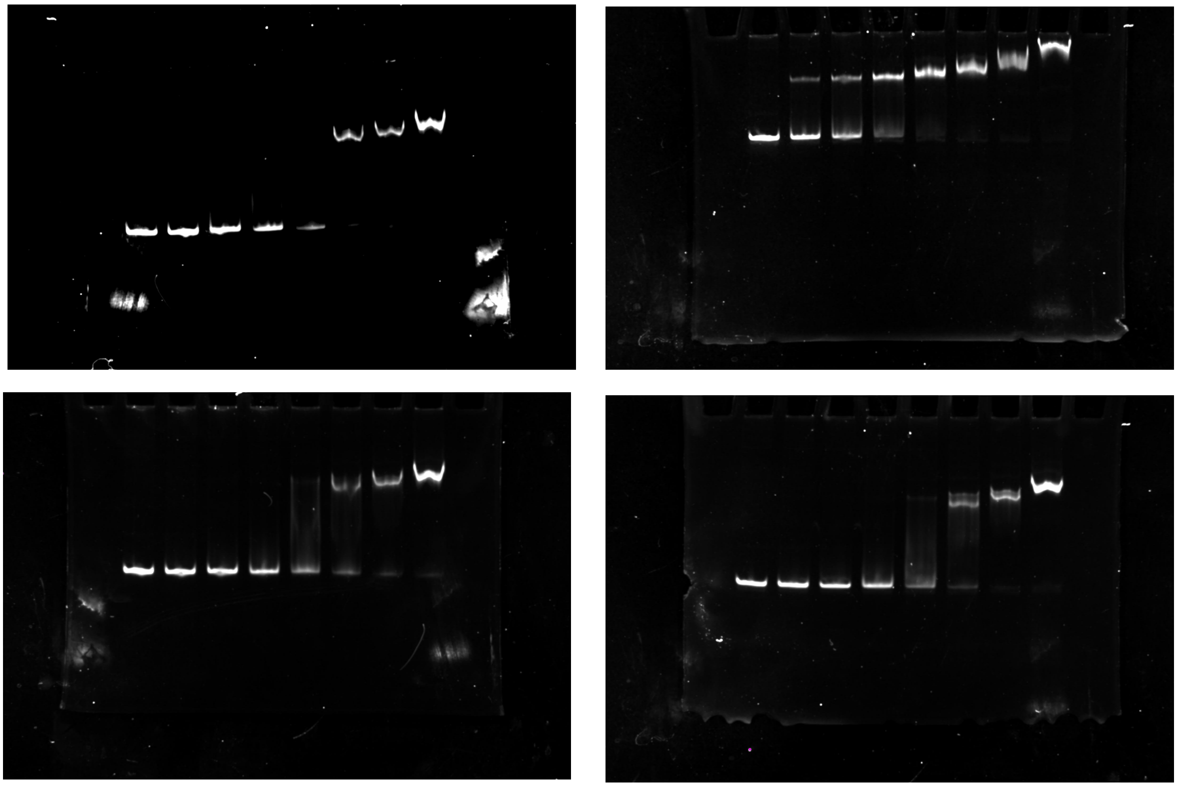

Supplementary Figure 4c (left) and 4d (right)

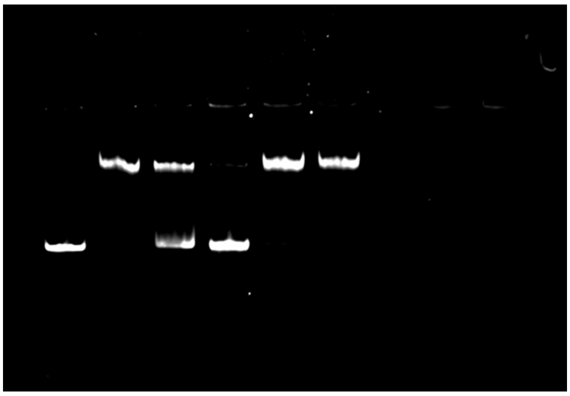

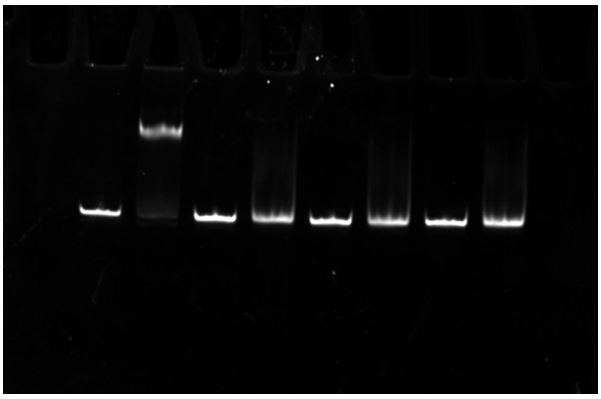

Supplementary Figure 8b

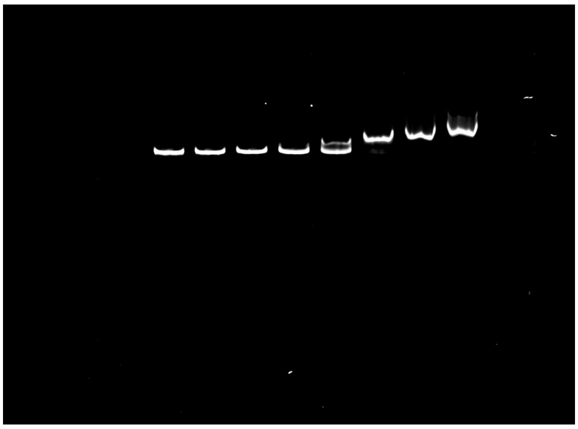

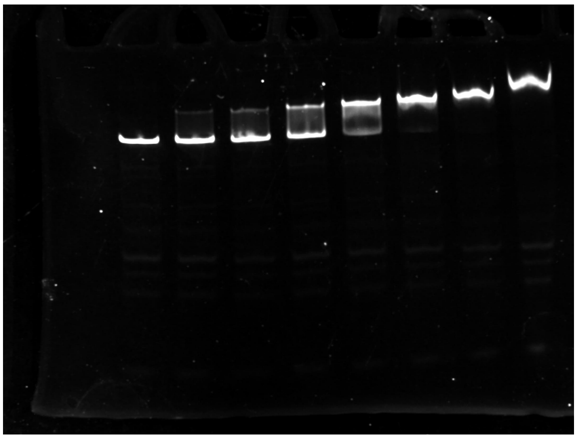

Supplementary Figure 8c

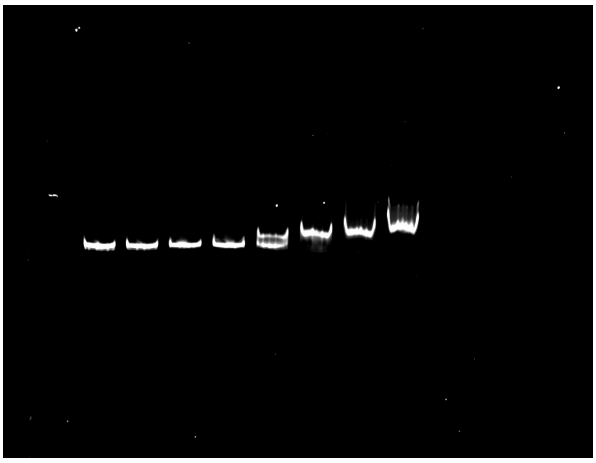

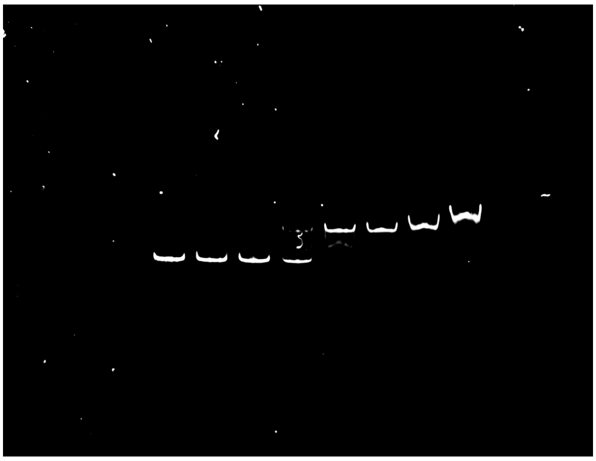

Supplementary Figure 8d (left) and 8e (right)

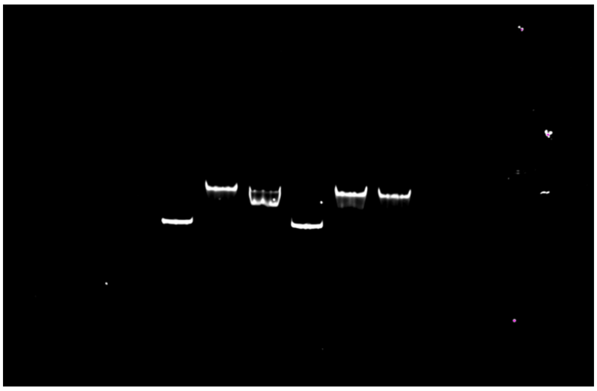

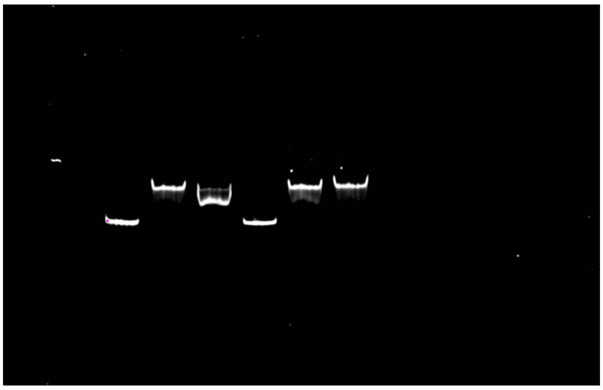

Supplement: Supplementary file 1 — Supplementary Information [file 41467_2024_45343_MOESM1_ESM.pdf]
