# Supplementary material for: TcrXY is an acid-sensing two-component transcriptional regulator of Mycobacterium tuberculosis required for persistent infection
Source: Nat Commun. 2024 Feb 22;15:1615. doi: 10.1038/s41467-024-45343-7 (PMC10883919; doi:10.1038/s41467-024-45343-7)
Supplement: Supplementary file 3 — Description of Additional Supplementary Files [file 41467_2024_45343_MOESM3_ESM.pdf]

Description of Additional Supplementary files

Supplementary Data 1: Differentially regulated genes in tcrXY-KO mutant compared to WT Mtb at pH 5.4 and pH7.0

Supplementary Data 2: Proteins of significantly altered abundance in CRISPRi induced strains cultured with ATc (+) compared to without (−) at pH7.0

Supplementary Data 3: Proteins of significantly altered abundance in CRISPRi induced strains cultured with ATc (+) compared to without (−) at pH5.4

Supplementary Data 4: GO enrichment analysis of the Mtb proteome upon CRISPRi silencing of Rv3706c and/or Rv3507A at pH7.0

Supplementary Data 5: GO enrichment analysis of the Mtb proteome upon CRISPRi silencing of Rv3706c and/or Rv3507A at pH5.4

Supplementary Data 6: Oligonucleotides used in this study
