# Supplementary material for: TcrXY is an acid-sensing two-component transcriptional regulator of Mycobacterium tuberculosis required for persistent infection
Source: Nat Commun. 2024 Feb 22;15:1615. doi: 10.1038/s41467-024-45343-7 (PMC10883919; doi:10.1038/s41467-024-45343-7)
Supplement: Supplementary file 10 — Reporting Summary [file 41467_2024_45343_MOESM10_ESM.pdf]

nature portfolio

Corresponding author(s): Nicholas P. West
Last updated by author(s): 2023/11/23

Reporting Summary

Nature Portfolio wishes to improve the reproducibility of the work that we publish. This form provides structure for consistency and transparency in reporting. For further information on Nature Portfolio policies, see our Editorial Policies and the Editorial Policy Checklist.

Statistics

For all statistical analyses, confirm that the following items are present in the figure legend, table legend, main text, or Methods section.

n/a	Confirmed	
☐	☒	The exact sample size (*n*) for each experimental group/condition, given as a discrete number and unit of measurement
☐	☒	A statement on whether measurements were taken from distinct samples or whether the same sample was measured repeatedly
☐	☒	The statistical test(s) used AND whether they are one- or two-sided<br>*Only common tests should be described solely by name; describe more complex techniques in the Methods section.*
☐	☒	A description of all covariates tested
☐	☒	A description of any assumptions or corrections, such as tests of normality and adjustment for multiple comparisons
☐	☒	A full description of the statistical parameters including central tendency (e.g. means) or other basic estimates (e.g. regression coefficient) AND variation (e.g. standard deviation) or associated estimates of uncertainty (e.g. confidence intervals)
☐	☒	For null hypothesis testing, the test statistic (e.g. *F*, *t*, *r*) with confidence intervals, effect sizes, degrees of freedom and *P* value noted<br>*Give P values as exact values whenever suitable.*
☐	☒	For Bayesian analysis, information on the choice of priors and Markov chain Monte Carlo settings
☐	☒	For hierarchical and complex designs, identification of the appropriate level for tests and full reporting of outcomes
☒	☐	Estimates of effect sizes (e.g. Cohen's *d*, Pearson's *r*), indicating how they were calculated

Our web collection on statistics for biologists contains articles on many of the points above.

Software and code

Policy information about availability of computer code

Data collection	Proteomic data (MS) was acquired using SCIEX OS (v2.1.6).<br>The code bases that support the collection of data are available on a GitHub repository: (https://github.com/FordeGenomics/TcrXY_Code). All codes and scripts used for data collection were uploaded to a TcrXY Code_repository, with the identifier doi:10.5281/zenodo.10157743
Data analysis	mage analysis for confocal microscopy was performed in Imaris (v9.6.1).<br>Peptides were identified using DIA-NN (v1.8)<br>The abundance of peptides and proteins were determined using PeakView (v2.1; SCIEX)<br>For statistical comparisons, the PeakView output was reformatted with a custom python script for use with MSstats (https://github.com/bschulzlab/reformatMS)<br>Differential protein abundance was compared using a mixed linear model using MSstats (v2.4)<br>Functional enrichment analysis was performed using the online Gene Ontology (GO) resource (http://geneontology.org)<br>Proteins associated with significantly enriched GO terms were visualised using STRING (v11.5)<br>Differential gene expression analysis was performed using DESeq2 (v1.40.2)<br>The RNAseq analysis pipeline used in this study is publicly available (https://usegalaxy.org.au/u/mstup1/w/rnaseq-analysis).<br>All custom script is declared in Code Availability section with code made available in Supplementary Information.

For manuscripts utilizing custom algorithms or software that are central to the research but not yet described in published literature, software must be made available to editors and reviewers. We strongly encourage code deposition in a community repository (e.g. GitHub). See the Nature Portfolio guidelines for submitting code & software for further information.

Data

Policy information about availability of data

All manuscripts must include a data availability statement. This statement should provide the following information, where applicable:

- Accession codes, unique identifiers, or web links for publicly available datasets
- A description of any restrictions on data availability
- For clinical datasets or third party data, please ensure that the statement adheres to our policy

The genome assemblies and sequencing datasets supporting the conclusions from this article have been deposited Sequence read data has been deposited to the SRA under the Bioproject accession PRJNA952396 and the SRA accessions SRR24208443-SRR24208453 and SRR24187079-SRR24187080. The mass spectrometry proteomics data presented in this study have been deposited to the ProteomeXchange Consortium (http://proteomecentral.proteomexchange.org) via the PRIDE partner repository with the dataset identifier PXD041697. The code bases that support the findings of this study are available on a GitHub repository: (https://github.com/FordeGenomics/TcrXY_Code). All codes and scripts were uploaded to a TcrXY Code_repository, with the identifier doi:10.5281/zenodo.10157743

Research involving human participants, their data, or biological material

Policy information about studies with human participants or human data. See also policy information about sex, gender (identity/presentation), and sexual orientation and race, ethnicity and racism.

Reporting on sex and gender	N/A
Reporting on race, ethnicity, or other socially relevant groupings	N/A
Population characteristics	N/A
Recruitment	N/A
Ethics oversight	N/A

Note that full information on the approval of the study protocol must also be provided in the manuscript.

Field-specific reporting

Please select the one below that is the best fit for your research. If you are not sure, read the appropriate sections before making your selection.

☒ Life sciences ☐ Behavioural & social sciences ☐ Ecological, evolutionary & environmental sciences

For a reference copy of the document with all sections, see nature.com/documents/nr-reporting-summary-flat.pdf

Life sciences study design

All studies must disclose on these points even when the disclosure is negative.

Sample size	No sample size calculations were made, however we determined the sample sizes used which are in line with discipline expectations while remaining conscious and sensitive to ethical consideration for the use of animals in scientific research. The sample sizes used achieved all of these important considerations and have allowed for rigorous statistical assessment. In the TB literature there are a myriad of examples where these (~5 mice per group) are used. A recent example, is Zhag et al. Nat Commun. 2022 Apr 26;13(1):2255 (PMID: 35474308). This study used 4 mice/group. We have used 5 and 6 mice/group depending on experiment.
Data exclusions	no data were excluded from analysis
Replication	A large majority of in vitro experiments were performed as three independent biological replicates. On occasion 2 biological replicate experiments were sufficient to achieve statistical relevance due to very large number of sample/data points included within each experiment. Some animal experiments were not repeated in the interests of animal ethics and the use of animals for scientific research. However all animal studies contained sufficient sample size to be statistically rigorous.
Randomization	These aspects are not relevant to our study. Mouse lines used were age and sex matched. There was not a need to randomise these experimental animal groups due to the deliberate matching, of what are otherwise clonal animals.
Blinding	Blinding was not necessary in our work. We did not conduct research that is subject to group bias.

Reporting for specific materials, systems and methods

We require information from authors about some types of materials, experimental systems and methods used in many studies. Here, indicate whether each material, system or method listed is relevant to your study. If you are not sure if a list item applies to your research, read the appropriate section before selecting a response.

Materials & experimental systems

n/a	Involved in the study
☒	☐ Antibodies
☒	☐ Eukaryotic cell lines
☒	☐ Palaeontology and archaeology
☐	☒ Animals and other organisms
☒	☐ Clinical data
☒	☐ Dual use research of concern
☒	☐ Plants

Methods

n/a	Involved in the study
☒	☐ ChIP-seq
☐	☒ Flow cytometry
☒	☐ MRI-based neuroimaging

Animals and other research organisms

Policy information about studies involving animals; ARRIVE guidelines recommended for reporting animal research, and Sex and Gender in Research

Laboratory animals	Female C57BL/6 mice 8 weeks to approx. 24 weeks of age used. the following sentence has been included in the manuscript (Methods;Mice) All mice were maintained in a physical containment level 3 (PC3) facility at The University of Queensland, providing a 12 hour light/dark cycle, ambient temperature range of 22-24C, at 40-60% humidity.
Wild animals	the study did not involve wild animals
Reporting on sex	Female mice only were used in this study. Sex does not affect the outcome of infection studies with Mycobacterium tuberculosis. Use of female mice ensures animals are not housed separately unless necessary for animal welfare considerations.
Field-collected samples	Study did not contain samples collected from the field
Ethics oversight	The Animal Ethics Committee of the University of Queensland provided guidance and approval for all work undertaken, as stated in the manuscript.

Note that full information on the approval of the study protocol must also be provided in the manuscript.

Plants

Seed stocks	No plants or seeds used in this study.
Novel plant genotypes	*Describe the methods by which all novel plant genotypes were produced. This includes those generated by transgenic approaches, gene editing, chemical/radiation-based mutagenesis and hybridization. For transgenic lines, describe the transformation method, the number of independent lines analyzed and the generation upon which experiments were performed. For gene-edited lines, describe the editor used, the endogenous sequence targeted for editing, the targeting guide RNA sequence (if applicable) and how the editor was applied.*
Authentication	*Describe any authentication procedures for each seed stock used or novel genotype generated. Describe any experiments used to assess the effect of a mutation and, where applicable, how potential secondary effects (e.g. second site T-DNA insertions, mosiacism, off-target gene editing) were examined.*

Flow Cytometry

Plots

Confirm that:

☒ The axis labels state the marker and fluorochrome used (e.g. CD4-FITC).

☒ The axis scales are clearly visible. Include numbers along axes only for bottom left plot of group (a 'group' is an analysis of identical markers).

☐ All plots are contour plots with outliers or pseudocolor plots.

☐ A numerical value for number of cells or percentage (with statistics) is provided.

Methodology

Sample preparation	The cells analysed via flow cytometry were bacterial, in culture. Samples were fixed in 4% paraformaldehyde to inactivate

Sample preparation	the bacteria before analysing on a CytoFLEX flow cytometer (Beckman Coulter). The below questions are not relevant to our study.
Instrument	CytoFLEX flow cytometer (Beckman Coulter).
Software	*Describe the software used to collect and analyze the flow cytometry data. For custom code that has been deposited into a community repository, provide accession details.*
Cell population abundance	*Describe the abundance of the relevant cell populations within post-sort fractions, providing details on the purity of the samples and how it was determined.*
Gating strategy	Attempts were made to identify single bacterial cells with initial scatter gates, before raw fluorescence data collected.

☐ Tick this box to confirm that a figure exemplifying the gating strategy is provided in the Supplementary Information.
